# miR449a/SIRT1/PGC-1α Is Necessary for Mitochondrial Biogenesis Induced by T-2 Toxin

**DOI:** 10.3389/fphar.2017.00954

**Published:** 2018-01-05

**Authors:** Shijie Ma, Yurong Zhao, Jianwei Sun, Peiqiang Mu, Yiqun Deng

**Affiliations:** ^1^Guangdong Provincial Key Laboratory of Protein Function and Regulation in Agricultural Organisms, College of Life Sciences, South China Agricultural University, Guangzhou, China; ^2^Key Laboratory of Zoonosis of Ministry of Agriculture, South China Agricultural University, Guangzhou, China

**Keywords:** T-2 toxin, mitochondrial biogenesis, miR-449a, SIRT1, PGC-1α

## Abstract

T-2 toxin is one of the type A trichothecenes produced mainly by the *Fusarium* genus. Due to its broad distribution and highly toxic nature, it is of great concern as a threat to human health and animal breeding. In addition to its ribotoxic effects, T-2 toxin exposure leads to mitochondrial dysfunction, reactive oxygen species (ROS) accumulation and eventually cell apoptosis. We observed that mitochondrial biogenesis is highly activated in animal cells exposed to T-2 toxin, probably in response to the short-term toxic effects of T-2 toxin. However, the molecular mechanisms of T-2 toxin-induced mitochondrial biogenesis remain unclear. In this study, we investigated the regulatory mechanism of key factors in the ROS production and mitochondrial biogenesis that were elicited by T-2 toxin in HepG2 and HEK293T cells. Low dosages of T-2 toxin significantly increased the levels of both mitochondrial biogenesis and ROS. This increase was linked to the upregulation of SIRT1, which is controlled by miR-449a, whose expression was strongly inhibited by T-2 toxin treatment. In addition, we found that T-2 toxin-induced mitochondrial biogenesis resulted from SIRT1-dependent PGC-1α deacetylation. The accumulation of PGC-1α deacetylation, mediated by high SIRT1 levels in T-2 toxin-treated cells, activated the expression of many genes involved in mitochondrial biogenesis. Together, these data indicated that the miR449a/SIRT1/deacetylated PGC-1α axis plays an essential role in the ability of moderate concentrations of T-2 toxin to stimulate mitochondrial biogenesis and ROS production.

## Introduction

T-2 toxin is one of the type A trichothecenes that are produced by different *Fusarium* species (McLachlan et al., 1992; Torp and Langseth, 1999). Its high toxicity and broad distribution lead to both sublethal and lethal toxicosis in humans and animals (Smith et al., 1995; Bennett and Klich, 2003). It is well known that T-2 toxin strongly inhibits the synthesis of eukaryotic proteins, DNA and RNA (Suneja et al., 1983; Jeffery et al., 1984; Thompson and Wannemacher, 1990). In addition, it increases the intracellular level of reactive oxygen species (ROS) at an early stage after its entry into eukaryote cells (Bouaziz et al., 2008). T-2 toxin induces cell apoptosis, possibly mediated by a mitochondrial pathway, which has been considered an important mechanism of its toxic effects, although the exact mechanism has still to be determined (Shinozuka et al., 1997; Islam et al., 1998).

Mitochondria, as dynamic organelles, play important roles in cellular metabolism, adenosine triphosphate (ATP) production, ROS generation, cell apoptosis and calcium regulation, and they are also the major intracellular source for antiviral responses (Wallace, 2005; Moore and Ting, 2008; Mishra and Chan, 2014). Mitochondrial biogenesis is governed by a regulatory network, and among of it peroxisome proliferator-activated receptor gamma and coactivator 1 alpha (PGC-1α) plays a central role (Gerhart-Hines et al., 2007; Scarpulla, 2011). Its regulation in mitochondrial biogenesis mainly depends on the extent of the acetylation/deacetylation status of PGC-1α (Lagouge et al., 2006).

Mammalian sirtuin 1 (SIRT1), an NAD^+^-dependent deacetylase, regulates mitochondrial biogenesis and function (Lagouge et al., 2006; Ou et al., 2014). This regulation mainly depends on the cytoplasmic and mitochondrial distribution of SIRT1, although SIRT1 is mainly localized in the nucleus (Aquilano et al., 2013). The overexpression of SIRT1 promotes the deacetylation of PGC-1α, which is an activated state of PGC-1α in the activation process of mitochondrial biogenesis (Nemoto et al., 2005; Price et al., 2012). Considering the profound impact of SIRT1 on mitochondrial biogenesis, exploring the link between the regulation of SIRT1 and T-2 toxin exposure is a key issue in unveiling the molecular mechanism of mitochondrial biogenesis upregulation and functions induced by T-2 toxin.

MicroRNAs (miRNAs) are small non-coding RNAs that have been identified as transcriptional or post-transcriptional regulators in gene expression (Bartel, 2009). miRNAs regulate the expression of protein-coding genes by degrading mRNA or inhibiting translation (Zeng et al., 2003; Bagga et al., 2005). miRNAs also play critical roles in metabolic regulation and cellular processes, including cell growth, differentiation, senescence, and apoptosis (Jiang et al., 2008). More than 16 miRNAs modulate SIRT1 expression, among them miR-34a induces colon cancer apoptosis and promotes senescence in endothelial cells via the down-regulation of SIRT1 expression (Yamakuchi, 2012). miRNAs also regulate mitochondrial biogenesis by downregulating TFAM and Foxj3 during myocyte differentiation and skeletal muscle adaptation to physical exercise (Yamamoto et al., 2012).

Our previous study showed that T-2 toxin upregulated mitochondrial proteins, consequently leading to an increase in mitochondrial mass in chicken primary hepatocytes (Mu et al., 2013). This response indicated that animal cells might respond to T-2 toxin exposure in the short term by upregulating mitochondrial biogenesis to cope with the toxicity effects of mitochondrial dysfunction and oxidative stress. However, the molecular mechanisms of mitochondrial enhancement in T-2 toxin-treated cells need to be further addressed. We hypothesize that T-2 toxin inhibits specific miRNA production to increase the SIRT1 homeostasis level in cells, meanwhile, this miRNA-SIRT1 axis consequently plays an important role in regulating mitochondrial biogenesis and function.

In this study, we observed that T-2 toxin significantly enhances mitochondrial biogenesis and functions in treated HepG2 and HEK293T cells. This mitochondrial-level upregulation mainly depends on the higher SIRT1 levels in T-2 toxin-treated cells. The elevated level of SIRT1 induced by T-2 toxin in both cell types is mainly mediated by downregulation of the level of miR-449a. The overexpressed SIRT1 in the cellular response to T-2 toxin exposure does not alter the the protein level of PGC-1α but deacetylates PGC-1α more efficiently, resulting of the activation of the mitochondrial biogenesis in HepG2 and HEK293T cells. We conclude that the stepwise regulation of miR449a/SIRT1/deacetylated PGC-1α probably plays a self-defense role by inducing mitochondrial biogenesis to balance the ROS overproduction in the early-stage response to T-2 toxin exposure.

## Materials and methods

### Cell culture and reagents

The human hepatocarcinoma cell line HepG2 (ATCC, HB-8065) and the human embryonic kidney cell line 293T (HEK 293T) (ATCC, CRL-1573) were cultured in DMEM medium supplemented with 10% fetal bovine serum (all from Gibco BRL, Carlsbad, CA, USA). Cultures were maintained in a 5% CO_2_ humidified atmosphere at 37°C. T-2 toxin and 3-(4,5)-dimethylthiazol(-2-yl)-2,5-diphenyltetrazolium bromide (MTT) were purchased from Sigma-Aldrich (St. Louis, MO, USA). H_2_O_2_ was purchased from Qiangsheng Chemical (Jiangsu, China).

### Cell viability assay

The cytotoxicity of T-2 toxin in HepG2 and HEK293T cells was measured using the MTT assay. Both cell lines were seeded in 96-well plates (1 × 10^4^ cells per well) and incubated until the cells reached ~60% confluence. After 24 or 48 h exposure to T-2 toxin concentrations in the range of 0–300 nM, with DMSO (0.01%) as a negative control, MTT (Sigma-Aldrich) solution was added to each well with growth DMEM medium for a final concentration of 5 mg/mL and incubated for 4 h at 37°C. Thereafter, all supernatants were aspirated, and DMSO (150 μL/well) was added to dissolve the formazan crystals. Finally, the optical density was measured using a microplate reader (Bio-Rad Laboratories, Hercules, CA, USA) at 490 nm. Cell viability was expressed as a percentage relative to the negative control, which was assumed to be 100% viable. Using the MTT assay data, dose-response curves were plotted to obtain the half-maximal inhibitory concentration (IC_50_) values. This IC_50_ value (for assaying cell viability) was used as a reference in subsequent experiments. Experiments were performed in triplicate and independently repeated.

### Plasmid construction and transfection

The full-length SIRT1 gene (GenBank: NM_012238) was amplified from p413TEF-SIRT1 (Addgene plasmid 51746) using the primer pair -CGCGGATCCATGGCGGACGAGGCGG (forward) and–ATTTGCGGCCGCTGATTTGTTTGATGGATAGTTC (reverse) and then subcloned into the pLPCX vector (cytomegalovirus promoter; Takara Bio, Inc.) for the SIRT1 overexpression assay. HepG2 and HEK293T cells were cultured in DMEM medium supplemented with 10% FBS. Cells were removed by trypsinization and grown in monolayers in 6-well plates at a density of 6 × 10^5^ cells per well at 37°C in 5% CO_2_ for 1 day prior to transfection. The plasmids were transfected into HepG2 and HEK293T cells using Opti-MEM Reduced Serum Media with Lipofectamine 2000 (Invitrogen, Rockville, MD, USA) according to the manufacturer's instructions.

The shRNA-expressing H1 retroviral system was described previously (Brummelkamp et al., 2002). The RNA-mediated interference of SIRT1 was performed in HepG2 and HEK293T cells using the pSUPER.Retro.puro vector (Oligoengine) encoding the shRNA sequence. The target sequence was GTGGCAGATTGTTATTAAT.

### Analysis of mitochondrial DNA content

HepG2 and HEK293T cells were grown in monolayers in 6-well plates at a density of 4 × 10^5^ cells per well for 1 day prior to T-2 toxin treatment. HepG2 cells were cultured for 24 h with 8 and 16 nM T-2 toxin; HEK293T cells were exposed to 2 and 4 nM T-2 toxin for 48 h. Total cellular DNA was isolated using an Omega DNA Kit (Omega, USA) according to the manufacturer's instructions and quantified using a Nanodrop 2000 spectrophotometer (Thermo Scientific, Wilmington, DE, USA). The DNA quality was tested by 1% agarose gel electrophoresis. The mitochondrial DNA copy number was measured by reverse-transcription quantitative polymerase chain reaction (RT-qPCR) and the 2^−ΔΔ*CT*^ method. The mtDNA content was calculated relative to the expression of mtDNA (D-loop) normalized to a single-copy nuclear β-globin gene. The following primers were used for RT-qPCR analysis: for the β-globin gene (120 bp), -GCTTCTGACACAACTGTGTTCACTAGC (forward) and—CACCAACTTCATCCACGTTCACC (reverse); for mtDNA (D-loop, 122 bp), -CACCAGCCTAACCAGATTTC (forward) and—GGGTTGTATTGATGAGATTAGT (reverse) (Bi et al., 2010). We generated standard curves for both fragments and calculated their respective amplification efficiencies to test whether using the 2^−ΔΔ*CT*^ method was appropriate. RT-qPCR was performed on a Bio-Rad CFX96 qPCR detection system (Bio-Rad, Hercules, CA, USA), according to the manufacturer's instructions. Reactions were performed in 20 μL volumes containing SYBR Green I Dye (Promega) under the following conditions: denaturation at 95°C for 5 min followed by 40 cycles of 15 s at 95°C, 15 s at 60°C and 30 s at 72°C. All assays were performed in triplicate, 2 ng of DNA were used per reaction, and the experiment was repeated three times.

### Analysis and quantification of mRNA and miRNA expression

HepG2 and HEK293T cells were grown in monolayers in 6-well plates at a density of 4 × 10^5^ cells per well for 1 day prior to T-2 toxin treatment. The T-2 toxin treated cells and control cells were pelleted and washed once with phosphate-buffered saline (PBS). One milliliter Trizol solution (Invitrogen) was added directly to the harvested cells, and the RNA extraction procedure was performed as described in the manufacturer's instructions. The RNA quality was checked by 1% agarose gel electrophoresis. The first cDNA strand was synthesized using the PrimeScript™ RT reagent Kit (Takara). RT-qPCR was performed on a Bio-Rad CFX96 real-time PCR detection system (Bio-Rad, Hercules, CA, USA), according to the manufacturer's recommendations. Standard curves were generated for both fragments, and their respective amplification efficiencies were calculated to test whether using the 2^−ΔΔ*CT*^ method was appropriate. The mRNA expression levels were measured using RT-qPCR performed in 20 μL volumes containing SYBR Green I Dye (Promega). GAPDH was chosen as an internal control for RNA level normalization. The primer pair sequences used are listed in Table S1. The 2^−ΔΔ*CT*^ method was employed to determine the relative expression of the target genes normalized to GAPDH, and the experiments were repeated three times.

The miRNA was extracted using the miRcute miRNA Isolation Kit (Tiangen, Beijing, China). The mature miRNAs were quantified using the TaqMan Hairpin-it miRNA and U6 snRNA Normalization RT-PCR Quantitation Kit (Tiangen, Beijing, China). The reactions were performed in triplicate, and the experiment was repeated three times.

### Mitochondrial mass analysis

HepG2 and HEK293T cells were grown in monolayers in 24-well plates at a density of 4 × 10^4^ cells per well for 1 day prior to T-2 toxin treatment. After 24 h or 48 h exposure to T-2 toxin, cells grown on coverslips were fixed in 4% paraformaldehyde for 10 min at room temperature and permeabilized with PBS containing 0.1% Triton X-100 (Sigma). The primary antibody against the intact surface of mitochondria (MAB 1273, Millipore) was diluted in 5% FBS in PBS (1:300) and applied at room temperature for 45 min. After being rinsed with PBS three times and incubated for 40 min with anti-mouse secondary antibody (AlexaFluor 488) diluted in PBS (1:2000), the cells were washed three times in PBS, and the cell nuclei were stained with 1 ng/mL DAPI (Genview, USA) for 1 min. The coverslips were then fixed onto slides using Prolong Gold antifade reagent (Invitrogen, Rockville, MD, USA) and viewed under a Zeiss Axio Observer D1 fluorescence microscope (Zeiss, Göttingen, Germany) using 100 × magnification. The fluorescence intensity levels were assessed with the ImageJ software (NIH).

### Transmission electron microscopy analysis

Transmission electron microscopy imaging was examined using ultrathin sectioning (70 nm). The cultured cells were prefixed in 2.5% glutaraldehyde in 0.1 M sodium cacodylate buffer overnight at 4°C, washed in the same buffer and then postfixed in 1% osmium tetroxide overnight at 4°C. The cells were then washed in 0.1 M sodium cacodylate buffer, dehydrated in an ethanol series and infiltrated with PolyBed epoxy resin. Ultrathin sections stained with uranyl acetate and lead citrate at room temperature were examined under a transmission electron microscope (Netherlands FEI Tecnai 12).

### Western blot analysis

Cells were lysed directly in RIPA buffer containing 50 mM Tris-HCl (pH 7.4), 150 mM NaCl, 1 mM EDTA, 1% Triton X-100, 0.1% SDS and 1 mM phenylmethylsulfonyl fluoride (PMSF). The lysates were adjusted for protein concentration with a BCA Protein Assay Kit (Pierce). The lysate proteins (30 μg) were resolved by 10% SDS-PAGE and then transferred to PVDF membranes (Millipore). The membranes were blocked (5% milk powder in TBST) at room temperature for 1 h and incubated overnight at 4°C with specific antibodies against SIRT1 (1:500) (mouse monoclonal; clone 10E4 04-1557; Millipore), PGC-1α (1:1000) (clone 3G6 2178, Cell Signaling Technology and clone 4C1.3 ST1202, Millipore), acetylated lysine (1:1000) (9441, Cell Signaling Technology) and GAPDH (1:1000) (sc-47724, Santa Cruz). After incubation with the secondary antibodies (Cell Signaling Technology) the bands were visualized by enhanced chemiluminescence using ECL and captured on X-ray films.

### Immunoprecipitation

Cells were treated with T-2 toxin and lysed in ice-cold lysis buffer containing 50 mM Tris-HCl (pH 8.0), 150 mM NaCl, 0.5% NP-40, 1 mM DTT, and 1 mM PMSF supplemented with a cocktail of protease and phosphatase inhibitors. The lysates were clarified by centrifugation at 12,000 *g* for 5 min and subjected to immunoprecipitation. Then, 100 μg of protein extract was incubated with primary antibody for 3 h at 4°C, followed by a second incubation of 3 h at 4°C with protein (A+G) agarose (Beyotime Company, Shanghai, China). The beads were washed three times, solubilized in 40 μL of 2× SDS sample buffer and analyzed by western blot. The intensity of each band was quantified with the ImageJ software (NIH).

### Measurement of ROS production

To evaluate the T-2 toxin-induced ROS level, T-2 toxin-treated cells were collected by adding trypsin and washed twice with ice-cold PBS, then suspended in PBS at a concentration of approximately 1 × 10^6^ cells/mL. The suspensions were then briefly vortexed and incubated with the addition of 10 μM carboxy-H_2_DCFDA in the dark at 37°C for 30 min. After incubation, the samples were analyzed with an Accuri C6 Flow Cytometer (BD Bioscience). H_2_O_2_ was used as a positive control (Yang et al., 1999). Cells were treated with 600 μM or 200 μM H_2_O_2_ for 24 h. The data collected from 10,000 individual cells were analyzed using the BD Bioscience Accuri C6 software. All experiments were performed independently three times.

### Luciferase activity assay

Plasmids containing the predicted miR-449a target sequence in the 3′UTR of SIRT1 and the mutant miR-449a target sequence were cloned into the pmirGLO vector. Both cell lines were grown in monolayers in 24-well plates at a density of 4 × 10^4^ cells per well at 37°C in 5% CO_2_ for 1 day prior to transfection. The plasmids and miR-449a were transfected into HepG2 and HEK293T cells using Opti-MEM Reduced Serum Media with Lipofectamine 3000 (Invitrogen). After 36 h, the cells were lysed, and the luciferase activities were measured with the Dual-Luciferase Reporter Assay System (Promega) using a Turner Designs TD-20/20n luminometer (Promega) and normalized to Renilla luciferase activity. All experiments were repeated three times.

### Statistical analysis

For each experiment, three replicates were performed, and all data were expressed as the mean ± the SD. Comparisons among different groups were calculated with one-way analysis of variance (ANOVA) followed by Dunnett's test for more than two groups and Student's test for two groups. Values of *P* < 0.05 were considered statistically significant.

## Results

### Cytotoxicity of T-2 toxin

**Figure 1:**
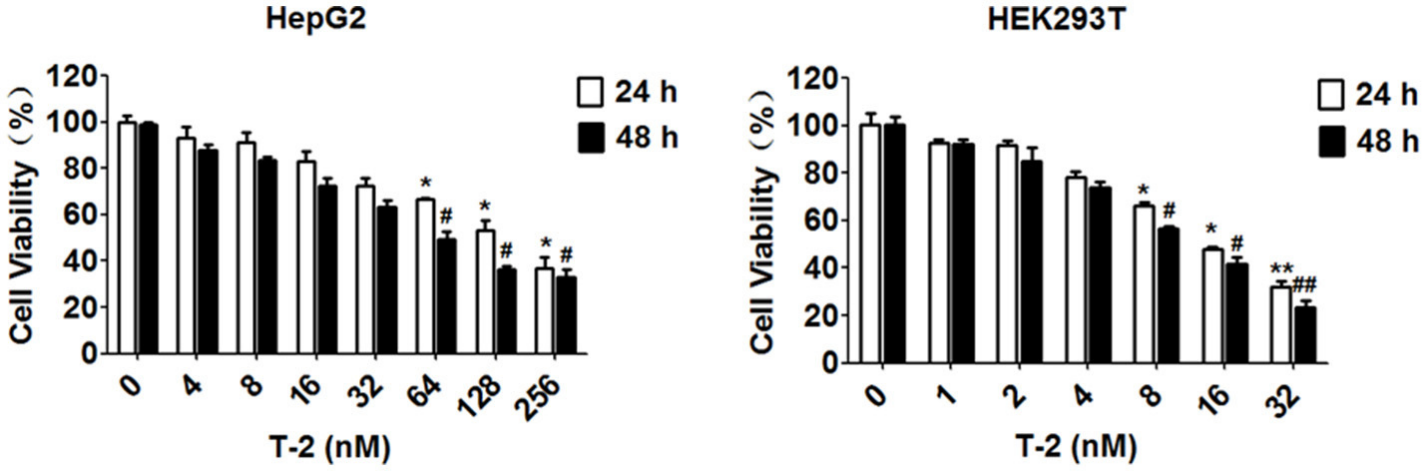
Cell viability assessment using an MTT assay. The IC_50_ value of T-2 toxin is 58.95 and 117.4 nM in HepG2 cells for 48 and 24 h, 10.51 and 15.65 nM in HEK293T cells for 48 and 24 h. ^*^P < 0.05 for comparison between the control and T-2 toxin-treated for 24 h groups. ^#^*P* < 0.05 for comparison between the the control and T-2 toxin-treated for 48 h groups.

The cell viability at the exposure of T-2 toxin has been assayed in many different cell lines by MTT assay with respect to the different IC_50_ values, such as 36.84 ± 7.17 ng/ml for human chondrocytic cell line (C28/I2), 17.51 ± 8.57 ng/ml for human hepatic epithelial cell line (L-02) and 72.76 ± 51.31 ng/ml for human tubular epithelial cell line (HK-2), respectively (Lei et al., 2017). In this study, we chose two human cell lines, HepG2 and HEK293T represent for T-2 toxin toxicosis in liver and kidney cells, and measure the IC_50_ value in these cell lines within 24 h and 48 h exposure. The MTT assay showed that the IC_50_ value of T-2 toxin, 58.95 and 117.4 nM in HepG2 cells for 48 and 24 h, 10.51 and 15.65 nM in HEK293T cells for 48 and 24 h, significantly reduced cell viability (Figure 1). On the basis of the MTT results, the T-2 toxin concentrations used in the subsequent experiments treated the cells with different T-2 toxin concentrations, are determined by maintaining 70–80% cell viability typically. It has been reported 44 ± 2.33% of HepG2 cells remains viable in 24 h treatment with 60 nM T-2 toxin (Bouaziz et al., 2008). Therefore, we determine the 24 h as the endpoint for exposure of T-2 toxin in HepG2 cells. Conclusively, unless otherwise specified, HepG2 cells were harvested and cultured for 24 h with 8 and 16 nM T-2 toxin, while HEK293T cells were exposed to 2 and 4 nM T-2 toxin for 48 h, followed by the determination of mitochondrial content, mitochondrial mass and ROS level.

### T-2 toxin exposure increases mitochondrial biogenesis

In our previous study, T-2 toxin increased oxidative stress and mitochondrial content in chicken primary hepatocytes, and comparative proteomics analysis revealed that many proteins related to oxidative stress, mitochondrial biogenesis or cell functions were significantly upregulated in T-2 toxin-treated chicken primary hepatocytes (Mu et al., 2013). We have measured the mitochondrial biogenesis under T-2 toxin treatment in four different cell lines, including HepG2, HEK293T, Hela and A549 cells. The increase in mitochondrial biogenesis was apparent in four cell lines while exposed to T-2 toxin. Moreover, HEK293T cells are the most sensitive to T-2 toxin and HepG2 cells are the most resistant to T-2 toxin. Therefore, we chose these two cell lines, HepG2 and HEK293T cells, as the representative cells to study the mechanisms of mitochondrial biogenesis induced by T-2 toxin.

**Figure 2:**
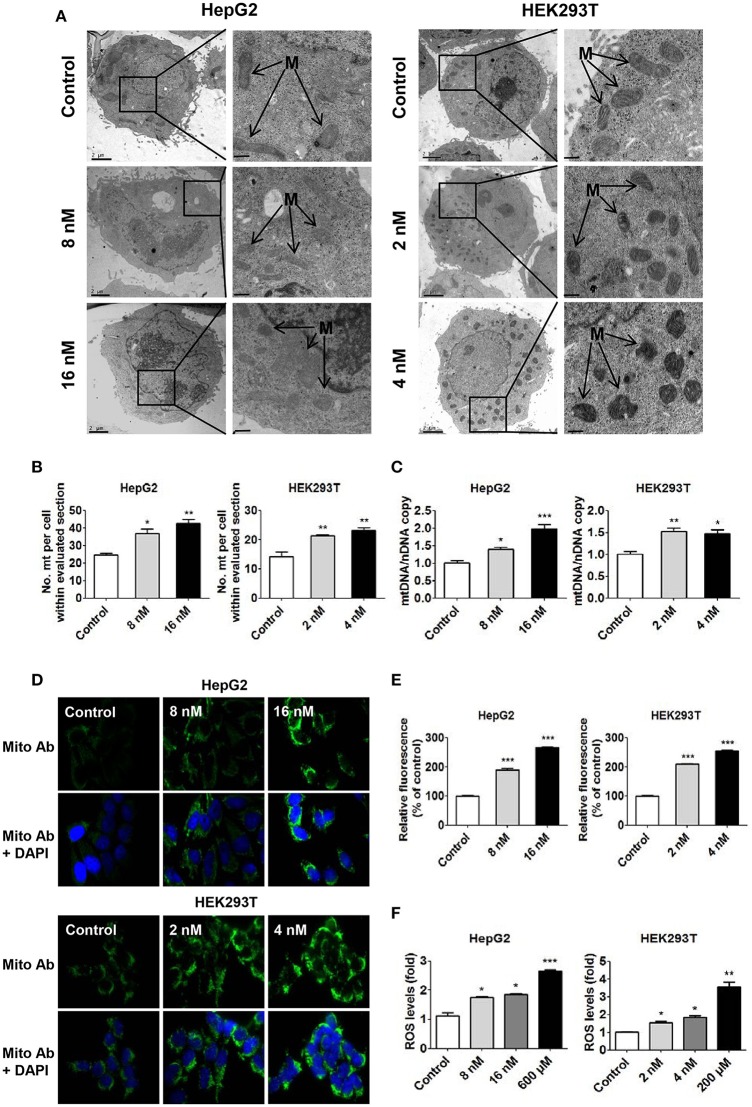
Increased mitochondrial biogenesis and ROS production in HepG2 and HEK293T cells in response to T-2 toxin treatment. **(A)** Representative transmission electron microscopy images of HepG2 and HEK293T cells treated with T-2 toxin. M indicates mitochondria. Scale bars: left panel, 2 μm; right panel, 1 μm. **(B)** Quantification of the number of mitochondria per cell in the imaged section (control, *n* = 35 cells; T-2 toxin treated, *n* = 35 cells). **(C)** Mitochondrial DNA content in HepG2 and HEK293T cells treated with T-2 toxin, analyzed by RT-qPCR. The primers used in this quantification are listed in section Materials and Methods. **(D)** Mitochondrial mass analysis by immunostaining. Mitochondrial staining (green) is shown in the top panels. To quantify the mitochondrial mass, treated cells were fixed and immunostained with anti-intact mitochondrial membrane antibody. Mitochondrial staining (green) and DAPI (blue) are shown in the bottom panels. Original magnification, 100×. The detailed measurement procedure is described in the section Materials and Methods. The fluorescence images for the GFP channel and the DAPI channel were taken with the same settings and exposure time. **(E)** Quantitative analysis of fluorescence intensity (mitochondrial staining) for HepG2 and HEK293T cells treated with T-2 toxin. Twenty-five images were analyzed for each count. **(F)** Detection of ROS generation in HepG2 and HEK293T cells treated with T-2 toxin and H_2_O_2_ stained with carboxy-H2DCFDA for 30 min at 37°C. Statistically significant differences are indicated by asterisks (^*^P < 0.05, ^**^P < 0.01, ^***^P < 0.001).

As mentioned above, in both cell lines, treatment with T-2 toxin at different concentrations led to a significant increase in mitochondrial content: 1.4-fold in the 8 nM T-2 toxin treatment and 1.6-fold in the 16 nM T-2 toxin treatment with respect to that in untreated HepG2 cells, and 1.5 and 1.6-fold in HEK293T cells treated with 2 and 4 nM T-2 toxin, respectively (Figures 2A,B). Quantitative analysis of mitochondrial length indicates that a 27% decrease under 8 nM T-2 toxin treatment and a 31% decrease under 16 nM T-2 toxin treatment in HepG2 cells. Similarly, a 28% decrease and a 29% decrease in mitochondrial length were observed in HEK293T cells treated with 2 and 4 nM T-2 toxin, respectively (Figures S1A,B). T-2 toxin clearly increased the mtDNA copy number, as measured by RT-qPCR: 39 and 98% more mtDNA was observed in HepG2 cells treated with 8 and 16 nM T-2 toxin for 24 h than in untreated HepG2 cells, and 52 and 48% more mtDNA was observed in HEK293T cells treated with 2 and 4 nM T-2 toxin for 48 h than in untreated HEK293T cells (Figure 2C). As shown in Figures 2D,**E**, T-2 toxin treatment significantly induced the upregulation of mitochondrial mass in HepG2 cells, resulting 1.9- and 2.7-fold higher mitochondrial mass relative to that in untreated HepG2 cells. Similar results were observed in HEK293T cells, with 2.1- and 2.6-fold increases in mitochondrial mass in T-2 toxin-treated cells with respect to untreated HEK293T cells. However, no significant increase of mitochondrial biogenesis was observed under 24 h T-2 toxin treatment in HEK293T cells (data not shown). Therefore, we chose 48 h T-2 toxin treatment for the subsequent experiments of HEK293T cells.

To confirm that the T-2 toxin-induced high mitochondrial level was related to the overproduction of ROS in both cells, we used carboxy-H2DCFDA as a probe to measure the level of ROS production in T-2 toxin-treated cells. According to the quantitative analysis of fluorescence intensity, T-2 toxin increased ROS production by 1.7- and 1.9-fold in HepG2 cells treated with 8 and 16 nM T-2 toxin and by 1.6- and 1.7-fold in HEK293T cells treated with 2 and 4 nM T-2 toxin, respectively (Figure 2F). As a positive control, H_2_O_2_-induced ROS production resulted in a 2.6-fold increase in ROS levels in HepG2 cells and a 3.6-fold increase in ROS levels in HEK293T cells (Figure 2F). Mitochondrial ROS was measured using a fluorescent probe MitoSOX (Invitrogen), T-2 toxin increased mitochondrial ROS by 1.9- and 2.2-fold in HepG2 cells treated with 8 and 16 nM T-2 toxin, and by 1.7- and 2.4-fold in HEK293T cells treated with 2 and 4 nM T-2 toxin, respectively (Figure S3). As a positive control, rotenone (Shanghai Macklin Biochemical Technology, China) increased mitochondrial ROS by 2.4- and 2.6-fold in HepG2 and HEK293T cells, respectively (Figure S2).

These mitochondria-related assessments proved that T-2 toxin can induce mitochondrial biogenesis and upregulate ROS production in human cells.

### T-2 toxin activation of Pgc-1α through upregulation of SIRT1

**Figure 3:**
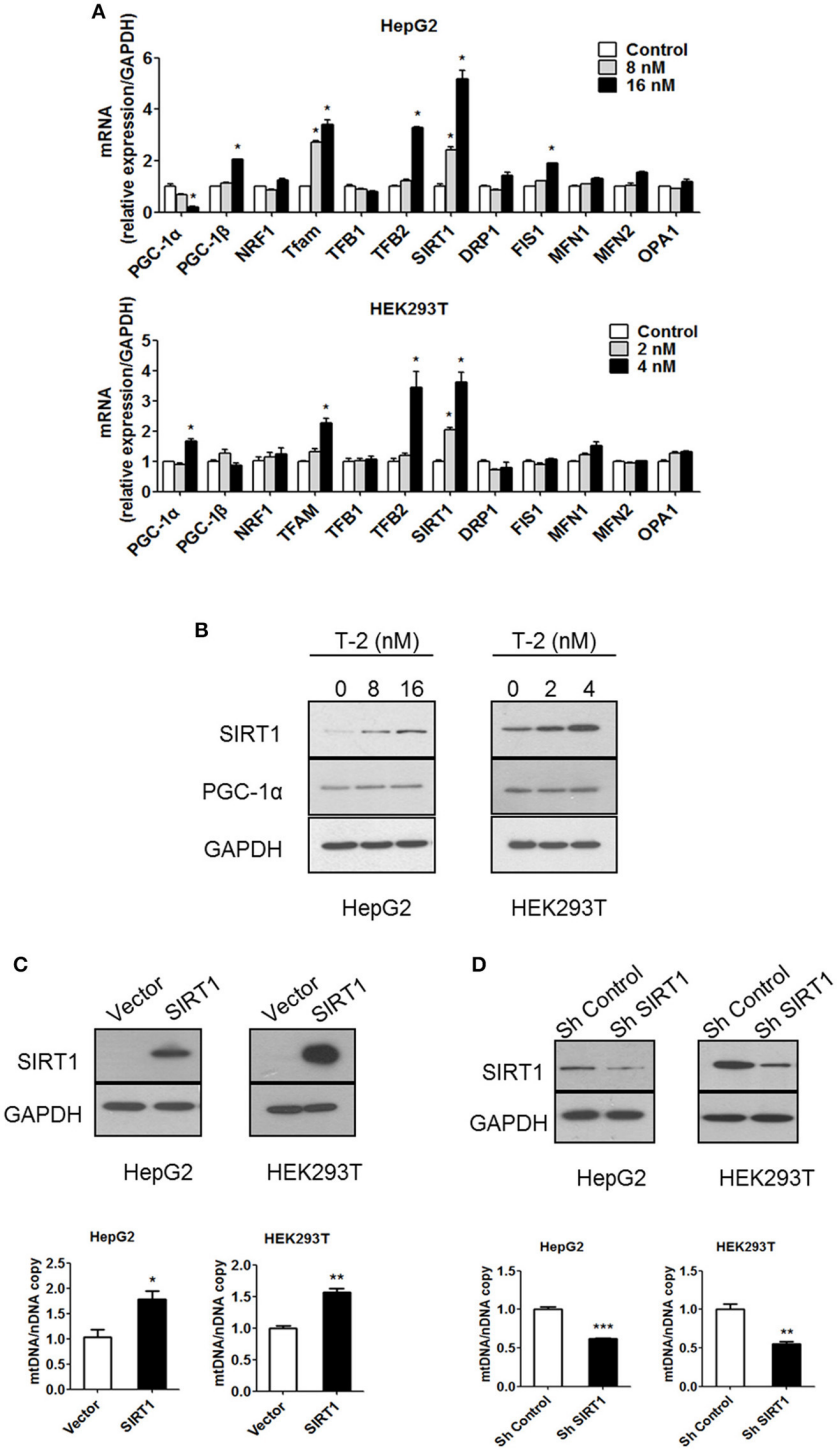
Low-dose exposure to T-2 toxin enhanced the mRNA and protein levels of SIRT1 in HepG2 and HEK293T cells. **(A)** PGC-1α, PGC-1β, NRF1, TFAM, TFB1, TFB2, SIRT1, DRP1, FIS1, MFN1, MFN2, and OPA1 mRNA levels in HepG2 (top) and HEK293T cells (bottom panel) were analyzed by RT-qPCR after T-2 toxin exposure at different doses. All the tested mRNA levels were normalized by the GAPDH mRNA level as the internal control. The experiments were repeated at least three times. **(B)** The protein levels of SIRT1, PGC-1α, and GAPDH in HepG2 (Left) and HEK293T cells (Right) with or without T-2 toxin treatment were assayed by western blotting with antibodies against SIRT1, PGC-1α, and GAPDH, respectively. The results shown are representative of three independent experiments. **(C)** SIRT1 expression level in HepG2 and HEK293T cells overexpressing SIRT1 was analyzed by western blotting. Mitochondrial DNA content in HepG2 and HEK293T cells overexpressing SIRT1 analyzed by RT-qPCR. **(D)** SIRT1 expression level in HepG2 and HEK293T cells infected with SIRT1 or nontargeting shRNA was analyzed by western blotting. Mitochondrial DNA content in HepG2 and HEK293T cells stably transfected with SIRT1-specific shRNA or nontargeting shRNA was analyzed by RT-qPCR. Statistically significant differences are indicated by asterisks (^*^P < 0.05, ^**^P < 0.01, ^***^P < 0.001).

Mitochondrial biogenesis requires coordinated control of the expression of numerous metabolic genes from the genomic and mitochondrial DNA (mtDNA). Therefore, the expression levels of many key factors involved in mitochondrial biogenesis, fission and fusion were measured in T-2 toxin-treated cells using RT-qPCR. The mRNA level analysis indicated that the administration of T-2 toxin upregulated the expression of sirtuin 1 (SIRT1): SIRT1 mRNA was increased by 2.4- and 5.2-fold relative to its level in the untreated HepG2 control. The same induction of high expression of mitochondrial transcription factor A (TFAM) and mitochondrial transcription factor B2 (TFB2) was also observed, with 2.7- and 3.4-fold higher TFAM mRNA levels in T-2 toxin-treated HepG2 cells than in untreated cells and 1.2- and 3.3-fold higher TFB2 mRNA levels in T-2 toxin treated HepG2 cells. The mRNA of other important factors, such as PGC-1α, exhibited no significant increment under T-2 toxin treatment (Figure 3A). The same patterns of induction of the expression of SIRT1, TFAM and TFB2 by T-2 toxin treatment for 48 h were also observed in HEK293T cells: 2.0- and 3.6-fold increases in the mRNA of SIRT1, 1.3- and 2.3-fold increases in TFAM, 1.2- and 3.5-fold increases in TFB2, and 0.9- and 1.6-fold increases in PGC-1α in comparison with untreated cells (Figure 3A). Key factors involved in mitochondrial fission and fusion were also analyzed. Except FIS1 which increased by 1.9-fold under T-2 toxin treatment in HepG2 cells (Figure 3A), the expression of other key factors analyzed, included DRP1, MFN1, MFN2, and OPA1, were not changed in HepG2 and HEK293T cells under T-2 toxin treatment (Figure 3A).

SIRT1 plays an important regulatory role in mitochondrial biogenesis by activating PGC-1α (Lagouge et al., 2006; Price et al., 2012). We assessed the protein levels of SIRT1 and PGC-1α by western blot. The results indicated that the SIRT1 level was significantly increased after T-2 toxin treatment, but PGC-1α expression showed minimal change (Figure 3B). Further analysis revealed that the overexpression of SIRT1 increased the mtDNA copy number, similar to the effect in T-2 toxin-treated cells, resulting in 79 and 57% more mtDNA than in the HepG2 and HEK293T control cells (Figure 3C). The knockdown of SIRT1 by the stable expression of SIRT1-specific shRNA reduced the number of mtDNA copies to 62 and 55% of that in HepG2 and HEK293T control cells (Figure 3D). These results confirmed that SIRT1 plays a key role in mitochondrial biogenesis.

**Figure 4:**
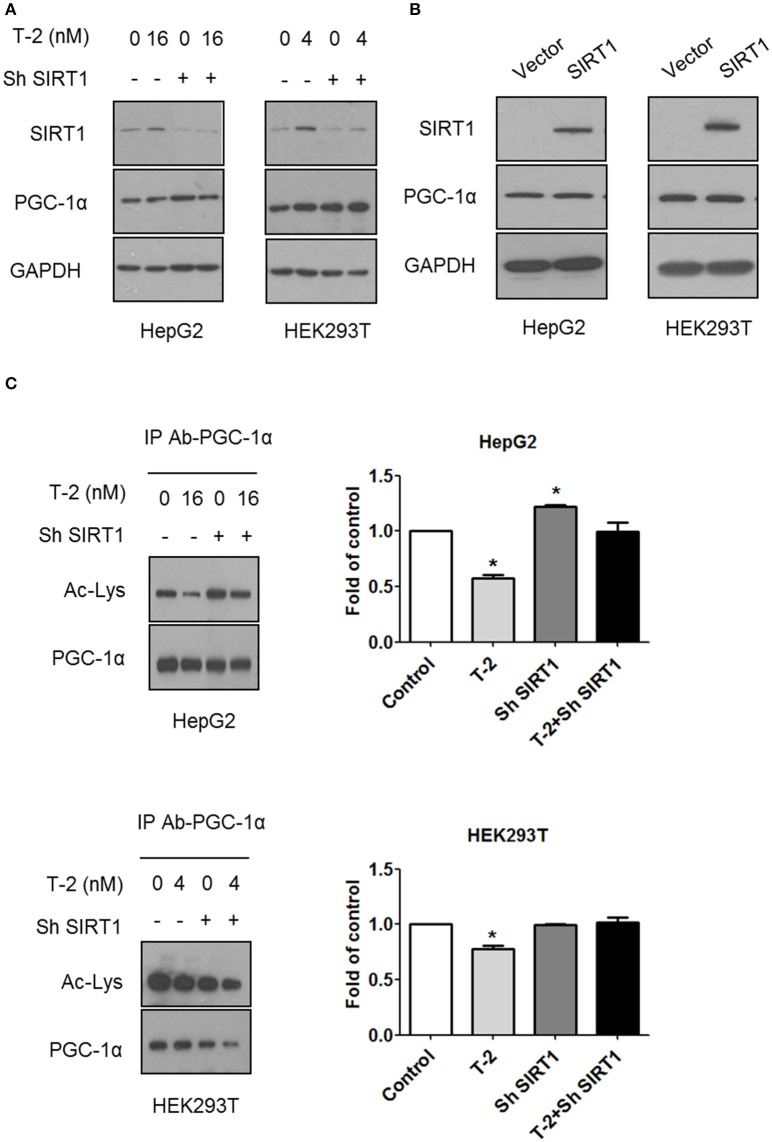
T-2 toxin induced PGC-1α deacetylation through SIRT1. **(A)** Representative immunoblot of SIRT1, PGC-1α, and GAPDH in T-2 toxin-treated cells stably transfected with SIRT1-specific shRNA or nontargeting shRNA. **(B)** Representative immunoblot of SIRT1, PGC-1α, and GAPDH in HepG2 and HEK293T cells overexpressing SIRT1. **(C)** HepG2 and HEK293T cells infected with SIRT1 or nontargeting shRNA were treated with T-2 toxin, and PGC-1α acetylation was tested by immunoprecipitation with an anti-PGC-1α antibody, followed by immunoblot analysis with the antibody specific to acetylated lysine. The data are expressed as fold values for three separate experiments. Statistically significant differences are indicated by asterisks (^*^P < 0.05).

PGC-1α plays an important role in establishing and modulating a network of transcriptional regulators related to mitochondrial biogenesis and function (Scarpulla, 2011). Treatment with T-2 toxin showed no effect on the expression of PGC-1α in cells with or without SIRT1 (Figure 4A). The overexpression of SIRT1 in HepG2 and HEK293T cells likewise did not affect the expression of PGC-1α (Figure 4B). These results suggested that the overexpression of SIRT1 probably activated PGC-1α by regulating deacetylation, instead of simply upregulating its protein levels. Therefore, we performed an immunoprecipitation (IP) experiment to assay the alterations in the acetylation modification of PGC-1α in HepG2 and HEK293T cells in response to T-2 toxin exposure. The analysis revealed that T-2 toxin significantly decreased the acetylation level of PGC-1α, whereas, this upregulated deacetylation of PGC-1α could not be observed in the SIRT1 knockdown cells with stably expressed shRNA for SIRT1, regardless of whether they were exposed to T-2 toxin or not (Figure 4C). These data suggested that SIRT1 is the main enzyme that deacetylates PGC-1α, and further, the overproduction of SIRT1 in T-2 toxin-treated cells elicited a strong increase in the deacetylation of PGC-1α.

### T-2 toxin increases mitochondrial biogenesis and ROS production in a SIRT1-dependent manner

**Figure 5:**
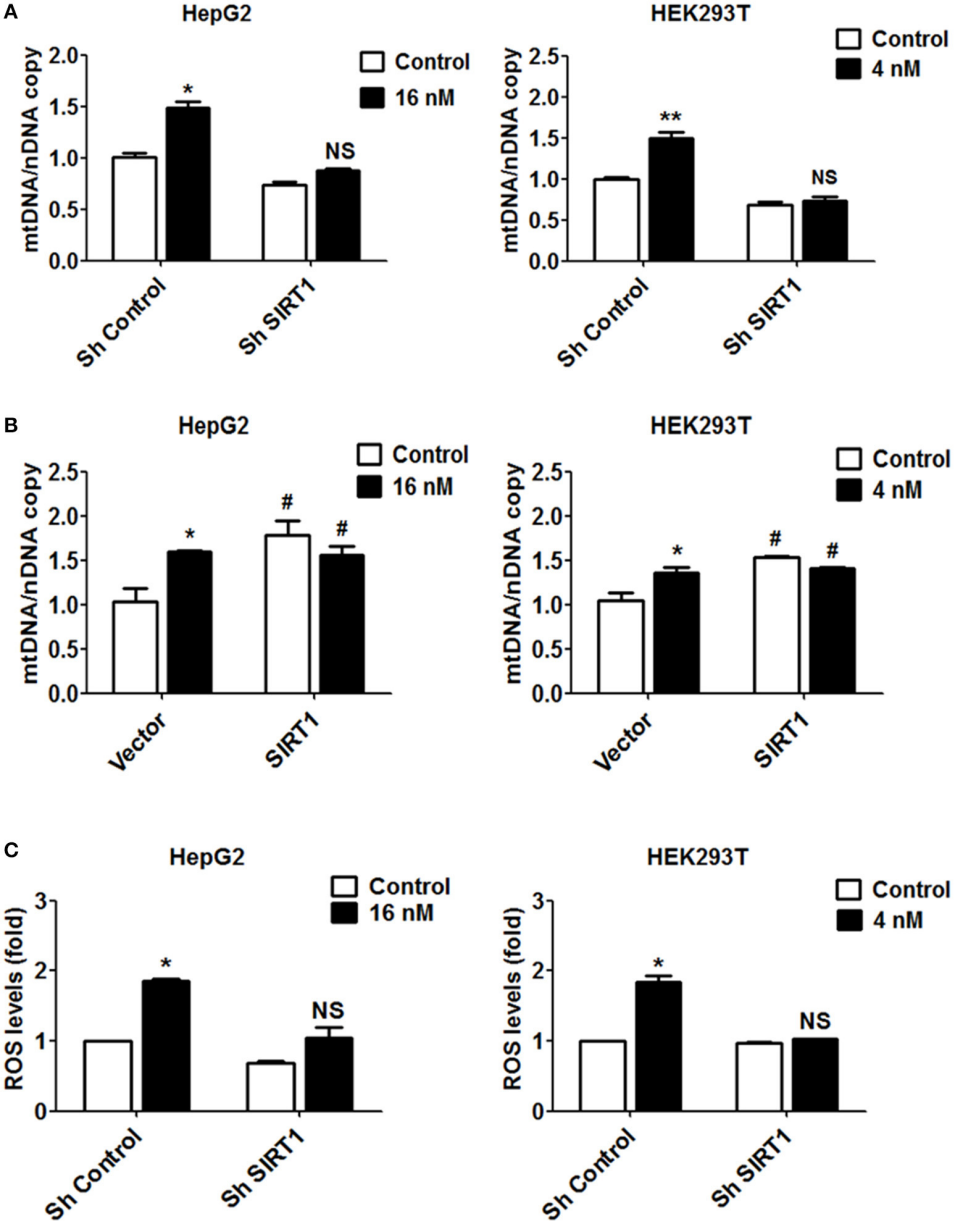
Increased mitochondrial biogenesis and ROS production in response to T-2 toxin treatment require SIRT1. **(A)** Mitochondrial DNA content analyzed in HepG2 and HEK293T cells with/without knockdown of SIRT1 exposed to T-2 toxin. Left, copies of mitochondrial DNA in HepG2 cells; Right, copies of mitochondrial DNA in HEK293T cells. **(B)** HepG2 and HEK293T cells overexpressing SIRT1 were treated with T-2 toxin, and the mitochondrial DNA content was determined using RT-qPCR. Left, copies of mitochondrial DNA in HepG2 cells; Right, copies of mitochondrial DNA in HEK293T cells. **(C)** ROS production in HepG2 and HEK293T cells infected with SIRT1 or nontargeting shRNA after T-2 toxin exposure was tested by a flow cytometry assay after staining with carboxy-H2DCFDA. Left, ROS levels measured in HepG2 cells; Right, ROS levels measured in HEK293T cells. ^*^P < 0.05 and ^**^P < 0.01 for comparison between the control and T-2 toxin-treated groups. ^#^*P* < 0.05 for comparison between the SIRT1 or SIRT1+T-2 toxin group and the control group, respectively.

While both the overexpression of SIRT1 and treatment with T-2 toxin have been shown to increase mitochondrial content, it remains to be established whether SIRT1 is always required for T-2 toxin to increase mitochondrial biogenesis in HepG2 and HEK293T cells. After treatment with T-2 toxin, cells showed a significant increase in mitochondrial biogenesis. The knockdown of SIRT1 reversed the increase in mtDNA copies in both types of cells treated by T-2 toxin, by 0.9-fold in HepG2 cells and 0.7-fold in HEK293T cells (Figure 5A). To further investigate the essential roles of SIRT1 in mtDNA production, we treated cells overexpressing SIRT1 with T-2 toxin to observe the mtDNA levels. The T-2 toxin treatment of SIRT1-overexpressing cells did not produce obvious differences from T-2 toxin treatment alone (Figure 5B), which suggested that the upregulation of mtDNA synthesis in T-2 toxin exposure mainly depend on the T-2 toxin-mediated overexpression of SIRT1. Next, we examined whether knockdown of SIRT1 could affect cell viability in HepG2 and HEK293T cells under T-2 toxin treatment. The MTT assay showed that the cell death increased in the SIRT1 knockdown cells under T-2 toxin treatment (Figures S3A,B).

Based on the observation that T-2 toxin treatment increased ROS level in both cell lines, we also evaluated the contribution of SIRT1 to ROS production. In the cells in which SIRT1 was downregulated by shRNA, T-2 toxin-induced ROS were apparently inhibited or abolished in comparison to their levels in the control cells treated with T-2 toxin. The ROS levels showed increases of 1.4-fold in *shSIRT1*-HepG2 cells, in contrast to 1.9-fold in HepG2 cells, and of 1.0-fold in *shSIRT1*-HEK293T cells, in contrast to 1.7-fold in HEK293T cells (Figure 5C). To analyze the effects of ROS on mitochondrial mass increase in response to T-2 toxin, HepG2 and HEK293T cells were pretreated with antioxidant MitoTEMPO (Sigma-Aldrich) for 1 h before T-2 toxin treatment. We observed that mitochondrial mass increase under T-2 toxin treatment was not obviously affected by pretreating with MitoTEMPO (Figure S4).

### T-2 toxin increases mitochondrial biogenesis through the miR-449a-SIRT1 axis

**Figure 6:**
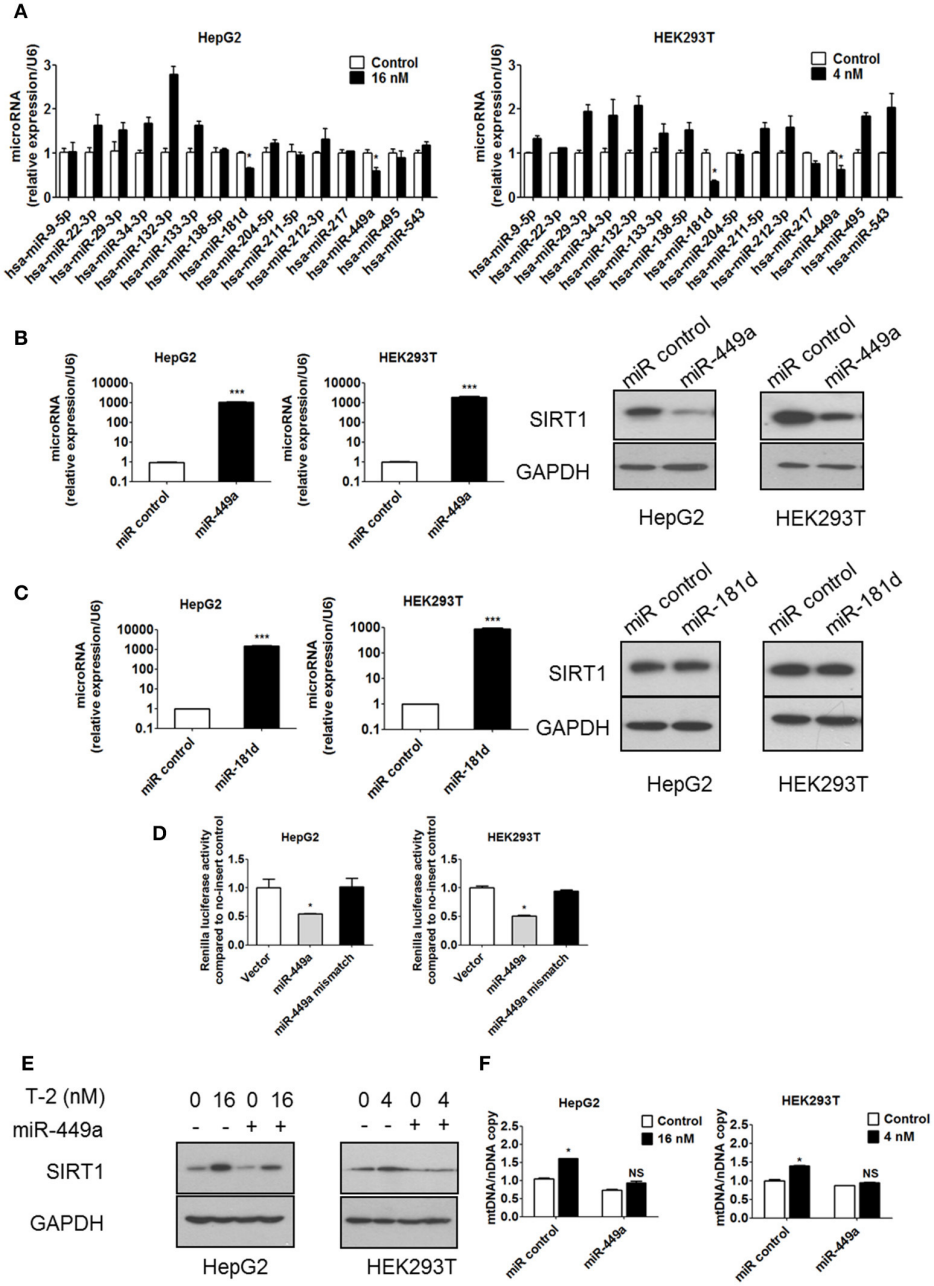
T-2 toxin enhances protein level of SIRT1 through miR-449a. **(A)** miRNA expression in T-2 toxin-treated cells. Top, miRNA levels in HepG2 cells analyzed by RT-qPCR; Bottom, miRNA levels in HEK293T cells analyzed by RT-qPCR. **(B)** The expression levels of miR-449a in HepG2 and HEK293T cells transfected with miR-449a mimics was detected by RT-qPCR. The protein level of SIRT1 in HepG2 and HEK293T cells transfected with miR-449a mimics was analyzed. Left, SIRT1 protein level in HepG2 cells; right, SIRT1 protein level in HEK293T cells. **(C)** The expression level of miR-181d in HepG2 and HEK293T cells transfected with miR-181d mimics was detected by RT-qPCR. The SIRT1 expression level in HepG2 and HEK293T cells transfected with miR-181d mimics was analyzed by western blotting. Left, SIRT1 protein level in HepG2 cells; Right, SIRT1 protein level in HEK293T cells. **(D)** Luciferase reporter assays were performed in HepG2 and HEK293T cells transfected with either the control pmirGLO vector or pmirGLO containing the SIRT1 3′UTR target sequence of miR-449a (both wild type and mutant), in the presence of miR-449a mimics. **(E)** The protein level of SIRT1 in HepG2 and HEK293T cells transfected with miR-449a mimics and treated with T-2 toxin was analyzed. **(F)** The mitochondrial DNA content in HepG2 and HEK293T cells transfected with miR-449a mimics and treated with T-2 toxin was analyzed. Statistically significant differences are indicated by asterisks (^*^P < 0.05, ^***^P < 0.001).

Our data have confirmed that the overproduction of SIRT1 in response to T-2 toxin exposure is the main reason for the upregulation of mitochondrial biogenesis and ROS levels. To unveil the molecular mechanisms of the T-2 toxin-induced overexpression of SIRT1, we assessed SIRT1 expression at the transcription level and the post-transcription level in T-2 toxin treated cells. We first excluded the transcriptional activation of SIRT1 expression by the construction of SIRT1 promoter-luciferase reporters in T-2 toxin-treated cells. Promoter analysis by the dual-luciferase assay showed that T-2 toxin induction in the promoter is limited, as no significant induction was observed (Figures S5A,B). Next, we addressed whether miRNAs were involved in the regulation of SIRT1 expression. The miRNA target prediction program miRanda (http://www.microrna.org/) was used to identify miRNAs that target the 3′ untranslated region (3′UTR) of SIRT1. The expression levels of the putative SIRT1-targeting miRNAs after in T-2 toxin treatment were determined by RT-qPCR and normalized with respect to the miRNA levels in untreated cells. Two putative miRNAs, miR-449a and miR-181d, were selected for further analysis because they were significantly downregulated by T-2 toxin, showing 0.6- and 0.7-fold changes in HepG2 cells relative to the untreated control and 0.6- and 0.3-fold changes in HEK293T cells (Figure 6A). Mature miRNAs suppress gene expression by binding to the 3′UTR of a specific mRNA, causing its degradation or translational inhibition. Based on this mechanism, we hypothesize that the downregulated miR-449a and miR-181d might release the tight suppression of SIRT1 expression in T-2 toxin treated cells. To evaluate the potential roles of miR-449a and miR-181d in SIRT1 expression, we transfected miR-449a and miR-181d mimics into HepG2 and HEK293T cells, respectively. In addition to remarkable changes in miR-449a expression, SIRT1 protein expression was significantly downregulated in the cells transfected with miR-449a mimics (Figure 6B), but no effect of miR-181d mimics transfection on SIRT1 protein expression was observed (Figure 6C). These data suggest that miR-449a potentially suppressed SIRT1 expression. To further validate whether SIRT1 is a direct target of miR-449a in HepG2 and HEK293T cells, we transfected a luciferase fusion construct containing either the wild-type SIRT1 3′UTR or a mutant to which miR-449a cannot bind into both cell lines. We then co-transfected the HepG2 and HEK293T cells with miR-449a mimics and the corresponding control to observed their repression ability with the dual-luciferase assay. The results showed that miR-449a mimics significantly suppressed the luciferase activity of the wild-type reporter, but no repression was observed in the reporter with the mutated SIRT1 3′UTR (Figure 6D). These data suggested that miR-449a targets the 3′UTR of SIRT1 mRNA to regulate its expression.

The overexpression of miR-449a has been shown to decrease the protein level of SIRT1, and we observed that T-2 toxin can suppress the level of miR-449a and, thus, plays a role in the upregulation of SIRT1 expression in response to T-2 toxin exposure. To further confirm this role, we overexpressed miR-449a in the T-2 toxin-treated cells to validate whether it plays a key role in the upregulation of mitochondrial biogenesis via controlling the expression of SIRT1. The overexpression of miR-449a reversed the heightened expression of SIRT1 induced by T-2 toxin and led to no apparent increase in mtDNA copy number (Figures 6E,F). Next, we determined whether overexpression of miR-449a attenuated T-2 toxin-induced deacetylation of PGC-1α. Transfection of miR-449a mimics significantly inhibited the deacetylation of PGC-1α in HepG2 and HEK293T cells under T-2 toxin treatment (Figure S6). We also examined whether overexpression of miR-449a could affect cell viability in both cell lines under T-2 toxin treatment. The MTT assay showed that the cell death increased in the cells transfected with miR-449a mimics than in control cells exposed to T-2 toxin (Figures S7A,B), suggesting that mitochondrial biogenesis increase may be a cellular protective effect under T-2 toxin treatment.

## Discussion

T-2 toxin exhibits clear cytotoxicity in animal cells, including the inhibition of eukaryotic protein biosynthesis and toxic effects on the cell membrane and on cell proliferation (Gyongyossy-Issa et al., 1985; Thompson and Wannemacher, 1990; Dugyala et al., 1994). In addition, T-2 toxin-induced apoptosis has been considered to be an important mechanism of its toxic effects (Zhuang et al., 2013). In particular, T-2 toxin led to the accumulation of ROS and was consequently believed to disturb the biogenesis and functions of mitochondria. In agreement with these results, we observed that the ROS production in T-2 toxin-treated HepG2 and HEK293T cells was obviously increased compared with the untreated cells. However, a previous study demonstrated that in chicken primary hepatocytes, T-2 toxin induces oxidative stress and the overproduction of mitochondria to cope with the accumulation of ROS, probably via the high levels of expression of the many proteins that are required for mitochondrial biogenesis (Mu et al., 2013). In this study, in both human cell lines, exposure to T-2 toxin resulted in a significant increase in the numbers of mitochondria as well as in the upregulation of mitochondrial DNA content and mass. Our observations suggested that cells can counteract the deleterious effects in the early stages of exposure to T-2 toxin by elevating the efficiency of mitochondrial biogenesis.

The pivotal protein in the control of mitochondrial biogenesis is PGC-1α (Wu et al., 1999; Lin et al., 2002; Puigserver and Spiegelman, 2003). It is well known that SIRT1 promotes mitochondrial biogenesis and function via a reduction in PGC-1α acetylation (Lagouge et al., 2006; Price et al., 2012). Although there is significant difference in the mRNA level of PGC-1α between T-2 toxin treated cells and the untreated controls, no change in the protein level. Instead we observed significantly increased deacetylation of PGC-1α in both cell lines under treatment with T-2 toxin. This result suggested that the acetylation modification of PGC-1α matters in the activation of mitochondrial biogenesis induced by T-2 toxin.

SIRT1 interacts directly with PGC-1α and thereby regulates the activity and acetylation status of PGC-1α (Nemoto et al., 2005; Rodgers et al., 2005). In further, the recent studies proposed that deacetylation by SIRT1 as a potential activator of PGC-1α transcriptional activity: deacetylated PGC-1α accumulates in cells with the high expression of SIRT1, accompanied by an increase in PGC-1α transcriptional activity, and subsequently enhances mitochondrial gene transcription and mitochondrial biogenesis (Lagouge et al., 2006; Gurd, 2011). Our study shows that T-2 toxin exposure increases the transcription and protein levels of SIRT1 in HepG2 and HEK293T cells. A significant increase in ROS levels was also observed in HepG2 and HEK293T cells treated with T-2 toxin (Figure S3). Mitochondria are the major ROS source and involved in the cell biological response by numerous cellular reactions (Kalogeris et al., 2014). It's possible that increased oxidative phosphorylation due to mitochondrial biogenesis leads to mitochondrial ROS production. These data indicated that SIRT1 potentially is a central regulator with positive effects on mitochondrial function and biogenesis in T-2 toxin exposure in human cells. The loss of function induced by *shSIRT1* alleviates the induction of mitochondrial biogenesis and ROS production by T-2 toxin. The overexpression of SIRT1 in both cell lines shows similar effects to those of T-2 toxin treatment, indicating that SIRT1 plays a pivotal role in the pathway. Knockdown and overexpression analysis proved that the control of SIRT1 expression has a key function in the determination of mitochondrial biogenesis and ROS production via its ability to deacetylate PGC-1α, especially in the event of T-2 toxin exposure. From these data, it can be concluded that T-2 toxin upregulates mitochondrial biogenesis and ROS production by decreasing the expression of acetylated PGC-1α in a SIRT1-dependent manner.

We also observed that SIRT1 overexpression during T-2 toxin exposure is mainly regulated by miRNAs instead of by transcription activation. miRNAs are known to suppress gene expression by either downregulating the levels of their target mRNAs or inhibiting mRNA translation (Bagga et al., 2005; Giraldez et al., 2006; Filipowicz et al., 2008). In T-2 toxin treatment, two miRNAs, miR-449a and miR-181d, have been found to be significantly downregulated via the screening of predicted miRNAs that potentially target the SIRT1 3′UTR. Further functional analysis confirmed that miR-449a plays the key role in SIRT1 expression. T-2 toxin suppresses miR-449a expression, which leads to elevated of SIRT1. The overexpression of miR-449a mimics suppresses the expression of its target, SIRT1, counteracts the induction effect of T-2 toxin in these cells, and subsequently blocks the increase in mitochondrial DNA content mediated by T-2 toxin. However, the exact mechanism of the suppression of miR-449a expression remains to be further investigated.

In this study, we proved that an early stage of T-2 toxin exposure exerted positive effects on mitochondrial biogenesis in human cells. Although it remains to be further addressed in animal models such as mice, pigs or chicken, this study is the first to report that SIRT1 is necessary for the induction of mitochondrial biogenesis by the exposure of relatively low dosages of T-2 toxin in human cells, and more importantly, this work explains the stepwise pathway induced by T-2 toxin. In this pathway, T-2 toxin suppresses miR-449a expression; the downregulation of miR-449a upregulates SIRT1 expression; the overexpression of SIRT1 leads to the increased accumulation of deacetylated PGC-1α; and the high accumulation of deacetylated PGC-1α activates mitochondrial biogenesis and ROS production in HepG2 and HEK293T cells.

## Author contributions

SM, YZ, JS, PM, and YD: conceived and interpreted the experiment; SM and YZ: performed the experiment and data analysis; SM: wrote the paper. PM: helped with editing and revising the manuscript. All authors approved the manuscript.

### Conflict of interest statement

The authors declare that the research was conducted in the absence of any commercial or financial relationships that could be construed as a potential conflict of interest.
