# Supplementary material for: miR449a/SIRT1/PGC-1α Is Necessary for Mitochondrial Biogenesis Induced by T-2 Toxin
Source: Front Pharmacol. 2018 Jan 5;8:954. doi: 10.3389/fphar.2017.00954 (PMC5760504; doi:10.3389/fphar.2017.00954)
Supplement: Supplementary file 1 [file DataSheet1.doc]

**SUPPLEMENTAL MATERIALS AND METHODS**

**Detection of Mitochondrial ROS**

HepG2 and HEK293T cells were grown in monolayers in 24-well plates at a density of 4 × 10^4^ cells per well for 1 day prior to T-2 toxin treatment. After 24 h or 48 h exposure to T-2 toxin, cells grown on coverslips were washed once with Hankʼs balanced salt solution (HBSS). 1μM Hoechst 33342 and 5 μM MitoSOX (Molecular Probes, Invirtrogen, USA) were added to the cells according to the manufacturer’s protocols. Fluorescence stained cells were imaged using a Zeiss Axio Observer D1 ﬂuorescence microscope (Zeiss, Göttingen, Germany) with 63× magniﬁcation. The fluorescence detection indicator setting for MitoSOX was excitation/emission maxima of approximately 510/580 nm. The ﬂuorescence intensity levels were assessed using the ImageJ software (NIH).

**Construction of Reporter Plasmids and Luciferase Activity Assay**

SIRT1 promoter constructs for luciferase assay were prepared as follows. A 2.1-kb fragment (−2147 to −1; +1 indicates the translational start site) of the 5’-ﬂanking region of SIRT1 (GenBank: NM_012238) was ampliﬁed from the genomic DNA of HepG2 cell. The PCR product was inserted into pGL3-Basic vector at *Sma* I and *Hind* III sites to generate the −2147-Luc construct. A series of 5’-promoter deletion constructs including −1029-Luc, −780-Luc, −736-Luc, −622-Luc, –589-Luc, −512-Luc, −297-Luc and −96-Luc were obtained on the basis of the −2147-Luc construct. All of the constructs are confirmed by sequencing. Primers for SIRT1 luciferase reporters are listed in Supplementary Table S2.

HepG2 cells were grown in monolayers in 24-well plates at a density of 5 × 10^4^ cells per well for 1 day prior to transfection. The cells in each well were transfected with 0.6 μg of the reporter construct and 0.06 μg of the pRL-TK control plasmid (Promega) with 2 μL of Lipofectamine 3000 (Invitrogen), according to the manufacturer's instructions. The cells were lysed after 24 h transfection and the luciferase activities were measured with the Dual-Luciferase Reporter Assay System (Promega) on a Turner Designs TD-20/20n luminometer (Promega). The amount of ﬁreﬂy luciferase activity was normalized to the amount of *Renilla* luciferase activity.

For analysis of inducible expression of SIRT1, T-2 toxin exposure experiments were performed. Prior to exposure experiments, the two groups were transfected with 0.6 μg of the reporter construct and 0.06 μg of the pRL-TK control plasmid in HepG2 cells. At 6 h after transfection, T-2 toxin was added to the treatment group. The cells were further cultured for 24 h and then luciferase activities were measured and normalized.

**FIGURE S1** The decrease of mitochondria length in HepG2 and HEK293T cells under T-2 toxin treatment. **(A)** Quantiﬁcation of the mitochondria length in HepG2 cells. **(B)** Quantiﬁcation of the mitochondria length in HEK293T cells (control, *n*=35 cells; T-2 toxin treated, *n*=35 cells). Statistically significant differences are indicated by asterisks (* P <0.05, ** P <0.01, *** P <0.001).

**FIGURE S2** The increase of mitochondrial ROS in HepG2 and HEK293T cells under T-2 toxin treatment. **(A)** Mitochondrial ROS of HepG2 cells stained by MitoSOX. **(B)** Quantification of mitochondrial ROS in HepG2 cells. **(C)** Mitochondrial ROS of HEK293T cells stained by MitoSOX. (D) Quantification of mitochondrial ROS in HEK293T cells. Rotenone was used as a positive control. Statistically significant differences are indicated by asterisks (* P <0.05, ** P <0.01, *** P <0.001).

**FIGURE S3** The cell viability assessment of SIRT1 knockdown cells under T-2 toxin treatment. **(A)** The cell viability of SIRT1 knockdown HepG2 cells at different doses of T-2 toxin treatment. **(B)** The cell viability of SIRT1 knockdown HEK293T cells at different doses of T-2 toxin treatment. Statistically significant differences are indicated by asterisks (* P <0.05).

**FIGURE S4** The mitochondrial mass changes of MitoTEMPO pretreated HepG2 and HEK293T cells under T-2 toxin treatment. **(A-D)** The mitochondrial mass increase in HepG2 cells under T-2 toxin treatment was not blocked by pretreating with MitoTEMPO (1 μM). **(E-H)** The mitochondrial mass increase in HEK293T cells under T-2 toxin treatment was not abrogated by pretreating with MitoTEMPO (10 nM). The HepG2 and HEK293T cells were pretreated with 1 μM and 10 nM MitoTEMPO for 30 min before T-2 toxin treatment, respectively. Statistically significant differences are indicated by asterisks (* P <0.05, ** P <0.01, *** P <0.001).

**FIGURE S5** Deletion analysis of the SIRT1 promoter in HepG2 cells. The schematic map of deletion constructs of SIRT1 promoter is shown on the left panel. The right panel shows the normalized ﬁreﬂy luciferase activity for each construct by the *Renilla* luciferase activity. **(A)** For analysis of basal expression of SIRT1. **(B)** For analysis of inducible expression of SIRT1, T-2 toxin exposure for 24 h with 16 nM. The results shown are representative of three independent experiments. Statistically significant differences are indicated by asterisks (* P <0.05, ** P <0.01, *** P <0.001).

**FIGURE S6** miR-449a blocked PGC-1α deacetylation induced by T-2 toxin. **(A-B)** The acetylation degree of HepG2 cells transfected with or without miR-449a mimics. **(C-D)** The acetylation degree of HEK293T cells transfected with or without miR-449a mimics. The miR-449a mimics transfected HepG2 and HEK293T cells were treated with T-2 toxin. Then, the PGC-1α was immunoprecipitated and the acetylation degree of which was analyzed by western blot using antibody against acetylated lysine. Statistically significant differences are indicated by asterisks (* P <0.05).

**FIGURE S7** The cell viability assessment in HepG2 and HEK293T cells transfected with miR-449a mimics under T-2 toxin treatment. **(A)** The cell viability of HepG2 cells transfected with miR-449a mimics at different doses of T-2 toxin treatment. **(B)** The cell viability of HEK293T cells transfected with miR-449a mimics at different doses of T-2 toxin treatment. Statistically significant differences are indicated by asterisks (* P <0.05).

**TABLE S1. The primer sequences used for RT-qPCR**.

| Gene name | Primer sequence | Accession numbers and references |
| --- | --- | --- |
| PGC-1α | F: CACCAAACCCACAGAGAACA  R: GGGTCATTTGGTGACTCTGG | NM_013261 |
| PGC-1β | F: CAGCCACTCGAAGGAACTTCA  R: CGGATGCTTGGCGTTCTG | NM_133263 |
| NRF1 | F: CGTTGCCCAAGTGAATTATTCTG  R: CCCTGTAACGTGGCCCAAT | NM_005011 |
| TFAM | F: GAAGTCGACTGCGCTCCC  R: ACTCCGCCCTATAAGCATC | NM_003201 |
| TFB1 | F: GGCAGAGAGACTTGCAGCCAA  R: CTTGAATGGCTGCTCTATCTTGGGC | NM_016020.3  This study |
| TFB2 | F: AACCGATCGGAGATTGGCTG  R: TTGTCACTTTCGAGCGCAAC | NM_022366.2  This study |
| SIRT1 | F: GCAACATCTTATGATTGGCACA  R: AAATACCATCCCTTGACCTGAA | NM_012238.4 |
| DRP1 | F: AATCTGAGGACATGGCACAG  R: CTTCACCAAAGATGAGTCTCCC | NM_012062.4  This study |
| FIS1 | F: TACCGGCTCAAGGAATACGAGA  R: TTGTCAATGAGCCGCTCCA | NM_016068.2 |
| MFN1 | F: TTGGAGCGGAGACTTAGCAT  R: TTCGATCAAGTTCCGGATTC | NM_033540 |
| MFN2 | F: ATGCATCC CCACTTAAGCAC  R: CCAGAGGGCAGAACT TTGTC | NM_014874.3 |
| OPA1 | F: GGCCAGCAAGATTAGCTACG  R: ACAATGTCAGGCACAATCCA | NM_015560.2 |
| GAPDH | F: CCTCAAGATCATCAGCAATGCC  R: TGGTCATGAGTCCTTCCACGAT | NM_002046.5 |

**TABLE S2. The primer sequences used for construction of reporter plasmids.**

| Primer | Sequence(5’- to 3’) |
| --- | --- |
| -2147 to -1 | F: TCCCCCGGGTATTGCATTCCACCAACGTAGCTG |
|  | R: CCCAAGCTTCTTCCAACTGCCTCTCTGGCC |
| -1029 to -1 | F: TCCCCCGGGGTACCCCTCGTTTTACATCTGG |
|  | R: CCCAAGCTTCTTCCAACTGCCTCTCTGGCC |
| -780 to -1 | F: TCCCCCGGGACACACGTTTGAAGCCAAGCT |
|  | R: CCCAAGCTTCTTCCAACTGCCTCTCTGGCC |
| -736 to -1 | F: TCCCCCGGGATCTCCAAACCTCCACGTCA |
|  | R: CCCAAGCTTCTTCCAACTGCCTCTCTGGCC |
| -622 to -1 | F: TCCCCCGGGGGAGAAAAGCAAGGAGCAGAA |
|  | R: CCCAAGCTTCTTCCAACTGCCTCTCTGGCC |
| -589 to -1 | F: TCCCCCGGGGAGGAGCTGTCAGAACGGTG |
|  | R: CCCAAGCTTCTTCCAACTGCCTCTCTGGCC |
| -512 to -1 | F: TCCCCCGGGACAGAACGACTATCCAACGTA |
|  | R: CCCAAGCTTCTTCCAACTGCCTCTCTGGCC |
| -297 to -1 | F: TCCCCCGGGAATTTGGCTGCACTACACGC |
|  | R: CCCAAGCTTCTTCCAACTGCCTCTCTGGCC |
| -96 to -1 | F: TCCCCCGGGTAAATCTCCCGCAGCCGGA |
|  | R: CCCAAGCTTCTTCCAACTGCCTCTCTGGCC |

**REFERENCES**

Benard, G., Trian, T., Bellance, N., Berger, P., Lavie, J., Espil-Taris, C., et al. (2013). Adaptative capacity of mitochondrial biogenesis and of mitochondrial dynamics in response to pathogenic respiratory chain dysfunction. *Antioxid Redox Signal* 19(4)**,** 350-365. doi: 10.1089/ars.2011.4244.

Bogacka, I., Xie, H., Bray, G.A., and Smith, S.R. (2005). Pioglitazone induces mitochondrial biogenesis in human subcutaneous adipose tissue in vivo. *Diabetes* 54(5)**,** 1392-1399.

Pal, A.D., Basak, N.P., Banerjee, A.S., and Banerjee, S. (2014). Epstein-Barr virus latent membrane protein-2A alters mitochondrial dynamics promoting cellular migration mediated by Notch signaling pathway. *Carcinogenesis* 35(7)**,** 1592-1601. doi: 10.1093/carcin/bgu069.

Sastre-Serra, J., Nadal-Serrano, M., Pons, D.G., Roca, P., and Oliver, J. (2012). Mitochondrial dynamics is affected by 17beta-estradiol in the MCF-7 breast cancer cell line. Effects on fusion and fission related genes. *Int J Biochem Cell Biol* 44(11)**,** 1901-1905. doi: 10.1016/j.biocel.2012.07.012.

Song, R., Xu, W., Chen, Y., Li, Z., Zeng, Y., and Fu, Y. (2011). The expression of Sirtuins 1 and 4 in peripheral blood leukocytes from patients with type 2 diabetes. *Eur J Histochem* 55(1)**,** e10. doi: 10.4081/ejh.2011.e10.

Zimmer, K.E., Montano, M., Olsaker, I., Dahl, E., Berg, V., Karlsson, C., et al. (2011). In vitro steroidogenic effects of mixtures of persistent organic pollutants (POPs) extracted from burbot (Lota lota) caught in two Norwegian lakes. *Sci Total Environ* 409(11)**,** 2040-2048. doi: 10.1016/j.scitotenv.2011.01.055.
